# Effects of different concentrations of artemisinin and artemisinin-iron combination treatment on Madin Darby Canine Kidney (MDCK) cells

**DOI:** 10.2478/v10102-012-0006-5

**Published:** 2012-03

**Authors:** Amir Ali Shahbazfar, Payman Zare, Hemn Mohammadpour, Hossein Tayefi-Nasrabadi

**Affiliations:** 1 Department of Pathobiology, Faculty of Veterinary Medicine, University of Tabriz, Tabriz, Iran; 2 Department of Basic Sciences, Faculty of Veterinary Medicine, University of Tabriz, Tabriz, Iran

**Keywords:** artemisinin, leishmaniasis, MDCK cell line, pathology, free radicals

## Abstract

Artemisinin is a sesquitrepenelactone with an endoperoxide bridge. It is a naturally occurring substance from *Artemisia* species plants. *Artemisia* species have been used in oriental medicine for centuries to treat malaria, gastrointestinal helminthosia, diarrhea, and as an antipyretic and sedative agent. Antileishmanial activity of the plants has been announced a few years ago. Dogs are the most important reservoir of leishmaniasis in some parts of the world. To use it as an antileishmanial drug in dogs, its side effects on different organs, among them the kidney as the organ of elimination have to be elucidated. Artemisinin with different concentrations (0.15, 0.3, 0.6 and 1.2 μg/ml) was added to the culture of MDCK (Madin darby canine kidney) cells with and without iron (86 μg/dl). All the changes were controlled and photographed every 12 hours using an invert microscope. After 60 hours, supernatants and cell extracts were examined for LDH (lactate dehydrogenase) concentration and total protein. Also TBARS (thiobarbituric acid reactive substances) test was performed on cell extracts. Some microscopic slides were prepared from the cells and stained with hematoxylin-eosin for microscopic exams. Biochemical parameters showed cellular reaction and injury in a concentration dependent manner. Cell injury was more severe in the iron-added groups. Microscopic exams showed cell and nuclear swelling, granular degeneration, vacuole and vesicle formation, cellular detachment, piknosis, karyorrhexis, cellular necrosis and inhibition of new mitosis. On using the drug for leishmaniasis treatment in the dog, it should be done with caution and supervision.

## Introduction

Leishmaniasis is made possible by a hemoflagellate protozoan species of the genus *Leishmania* (Ma *et al*., 2004). Rodents, dogs, sanguine marsupials, and other wild animals are carriers of the disease. In some areas of the world, dogs are the most important carriers. The disease is transferred by mosquitoes of the genera *Lutzomyia* and *Phlebotomus*. According to the World Health Organization (WHO), 88 countries are affected by leishmaniasis, with approximately 350 million people at risk of infection. With 12 million current cases, the occurrence of leishmaniasis is increasing worldwide with 1–2 million new cases reported each year despite attempts to fight the disease (de Carvalho & Ferreira, 2001). Leishmaniasis encompasses the following three clearly recognizable clinical manifestations: 1) generalized visceral infection (visceral leishmaniasis or “Kala-azar”), 2) cutaneous leishmaniasis (oriental button), and 3) mucocutaneous leishmaniasis (Rocha *et al*., 2005). The disease can be restrained chiefly by vector and reservoir control and by infected case management. One approach for management is drug therapy.

*Artemisia* species are pasture herbs in vast areas of the world. *Artemisia* species have been used for centuries in oriental medicine for treating malaria, gastrointestinal helminthiasis, diarrhea, and as antipyretic and sedative agents. Further research has led to extraction of Artemisinin from *Artemisia* spp. The substance is a sesquitrepenelactone with an endoperoxide bridge (Fishwick *et al*., 1995). A number of studies have been carried out in order to explore antiprotozoal activity in different species (Arab *et al*., 2006). Currently, its most important application is for the treatment of malarias that are resistant to Quinolones (Fishwick *et al*., 1995). Artemisinin contains a 1, 2, 4-trioxane ring and the highly reactive endoperoxide group plays a key role in its anti-malarial activity (Galasso *et al*., 2007). Reductive breaking of this bond, mediated by iron complexes, generates reactive radicals and the resultant chain of reactions damages the parasite (Galasso *et al*., 2007). Several mechanisms have been recommended for the anti-malarial effect of Artemisinin and other related endoperoxides. First, reductive cleavage of the endoperoxide bridge of Artemisinin occurs, followed by intramolecular electronic rearrangements that produce carbon-centered radicals. Subsequent reactions, including the alkylation of proteins, lead to the death of the parasite (Zhang & Gerhard, 2008). Artemisinin restores nitric oxide production in *Leishmania donovani* infected macrophages to the level of uninfected cells. The addition of Artemisinin causes a spurt in both IFN-? and IL-2 in Leishmania-infected CD3+ T lymphocytes, bringing their levels to those comparable with uninfected control cells (Sen *et al*., 2010). Some researchers tested different concentrations of *Artemisia* extract on *Leishmania* cultures and reported the effect of different concentrations (Sharif *et al*., 2006). Although Artemisinin was considered to be safe in therapeutic doses, a number of researchers have shown different side effects of the drug, as well as its synthetic or semi-synthetic derivatives in humans and some animal species. In animal studies, there has been clear evidence of embryolethality and some evidence for morphological abnormalities induced during early pregnancy, without maternal toxicity following drug consumption. Hormonal imbalance in progestagens and in testosterone levels occurred following Artemisinin administration during pregnancy in Wistar rats (Boareto *et al*., 2008).

One of the necessary steps prior to introducing a drug as a therapeutic agent for a particular disease, such as Artemisinin for Leishmaniasis, is a toxicity study and determination of the drug's pathologic effects in target animals. According to our literature review, there are no documented data regarding the toxicity of Artemisinin in MDCK cells. The anti-malarial action of artemisinin has been attributed to intra-parasitic iron- or heme-catalyzed cleavage of the endoperoxide bridge leading to the generation of toxic free radicals or intermediates (Efferth *et al*., 2004; Meshnick *et al*., 1993). Most of the drug effects have been attributed to dihydroArtemisinin (DHA), the active metabolite in the drug family. However, Artemisinin has some effects without being metabolized to DHA.

The aim of this study was to identify the pathologic and cytotoxic effects of Artemisinin using various concentrations in the presence or absence of an iron source on MDCK cells (Madin-Darby Canine Kidney cells), since the kidney is the major organ of drug elimination from the body.

## Methods

### The cells

MDCK cells were obtained in Eagle's minimal essential medium (MEM) with non-essential amino acids, 10% FBS (fetal bovine serum), without antibiotics, and cultivated at 37 °C in a humidified incubator with 5% CO_2_. MDCK cells were grown in 25 cm^2^ cell culture plastic flasks containing a RPMI 1640 medium (Roswell Park Memorial Institute medium) with a 10% fetal bovine serum. Media were refreshed every 72 hr until the cells entirely filled the bottom of the culture flask (7–10 days). Source of MDCK cell line: Pasteur institute, Tehran, Iran.

### Drug

Artemisinin, 99% pure, was obtained. Purification was tested with high performance liquid chromatography (HPLC). Artemisinin was dissolved in DMSO (dimethyl sulfoxide). A fresh innate source solution was produced for each treatment. Different amounts of the solution were added to cell cultures to achieve the desired concentration (0.15, 0.3, 0.6, and 1.2 μg/ml). Cell cultures were divided into four Artemisinin treatments, four Artemisinin and ferrous sulfate treatments (Combination therapy), one negative control group, and one iron control group. The concentration of iron was constant at 86 μg/dl (Seyrek *et al*., 2009). Iron was added as ferrous sulfate heptahydrate.

### Biochemical analysis

After 60 hours, the supernatant media from the individual cultures were recovered and centrifuged (5 000 rounds per minute for 5 minutes) in order to isolate floating cells. A total protein measurement and an enzyme analysis (LDH) was performed on the centrifuged supernatants and the cell fraction extracts. Total protein was measured by the method of Lowry (Lowry *et al*.,^1951^), with bovine serum albumin as standard. Lactate dehydrogenase activity of cells fraction and supernatant was measured by the method of Babson and Babson (Babson and Babson.,^1973^).

The total lipid peroxidation product, as indicated by malonyl dialdehyde (MDA) formation in the cell homogenate, was assayed using the thiobarbituric acid reactive substances (TBARs) method (Buege & Aust, 1978).

Cell fraction extracts were prepared by mixing centrifuged precipitants and half of the cells of flask bottoms that were removed using a plastic stir rod. Cells were cleaved using freeze-thaw cycles in a liquid nitrogen tank. Then all specimens were tested under light microscopy with a Neubauer slide for the presence or absence of uncleaved cells. For the last step, precipitants were removed after centrifugation of cleaved cells (Hackett *et al*., 1994; Satoh, 1978).

### Light microscopy

Cellular changes were monitored and photographed every 12 hours until the end of the exposure (60 hr) under an invert microscope. The experiment was repeated three times. After 60 hr, the rest of cell cultures were fixed with methanol and the plastic bottom of the flasks with fixed cells (cells that were not removed for extraction procedure) were stained with hematoxylin-eosin for microscopic analysis.

### Statistical analysis

Data from culture supernatants and cell fraction extracts were analyzed using SPSS (Statistical Package for Social Sciences, SPSS Software 16, SPSS Inc., NY, USA) software (Table 2 and 3). One way ANOVA (Analysis of Variances) and Tukey – as *post hoc* – was used (confidence interval =95%). The data from the two control groups and the two main treatment groups (Artemisinin and combination therapy) with the same amount of Artemisinin were compared, two by two, with an independent sample T-test.

## Results

### Pathology

**Figure 1:**
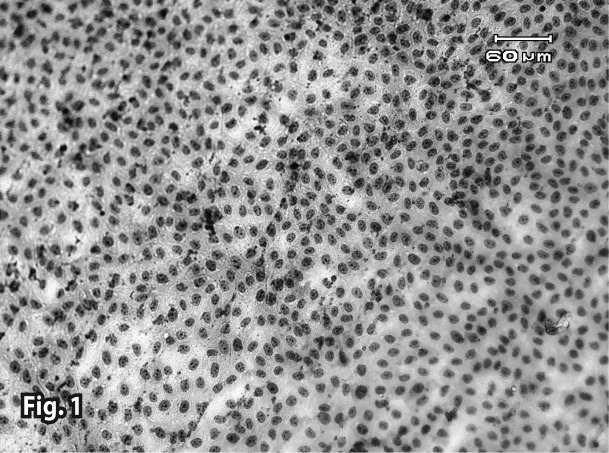
Normal cells in the control group (60^th^ hour). Invert microscope (bar=60μm).

**Figure 2:**
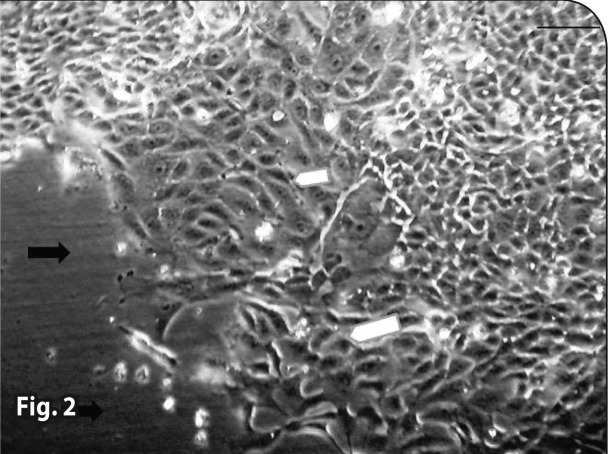
Normal cells in the control group (60^th^ hour). H&E staining.

**Figure 3:**
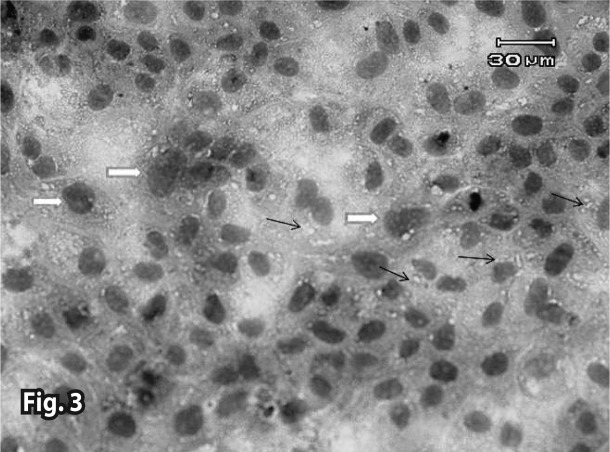
The area of denudation (black right arrows) and regeneration( white pentagons) in the 9×10^–2^ mg/ml Artemisinin group without iron (60^th^ hour). Invert microscope (bar=30 μm).

**Figure 4:**
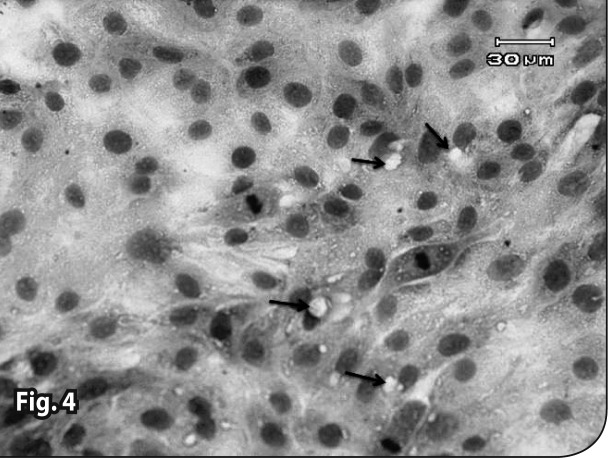
The granular degeneration and formation of small vacuoles (Arrows). Cell and nuclear swelling (Right arrows)in the 2.25×10^–2^ mg/ml Artemisinin group with Iron (36^th^ hour). H&E staining.

**Figure 5:**
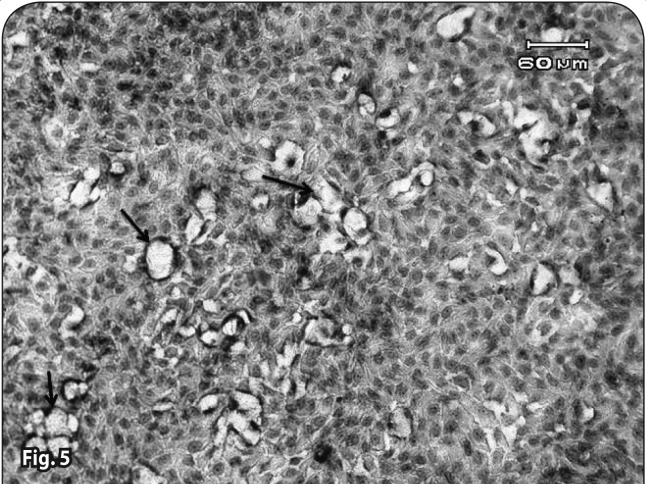
Cell and nuclear swelling and the beginning of large vacuole formation (Arrows) in the 4.5×10^–2^ mg/ml Artemisinin group without iron (48^th^ hour). H&E staining.

**Figure 6:**
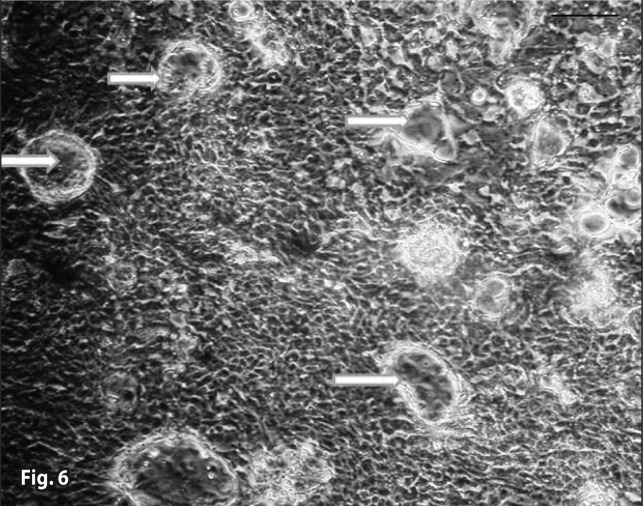
Severe vesicle formation (Arrows) in the 9×10^–2^ mg/ml Artemisinin group with iron (36^th^ hour). H&E staining.

**Figure 7:**
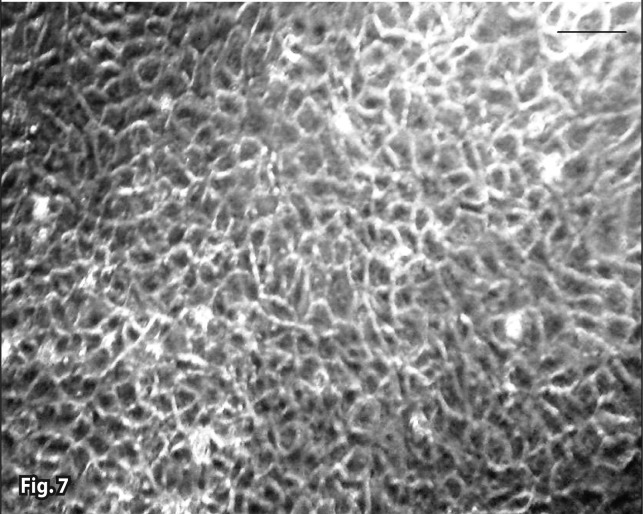
Severe vesicle formation (white right arrows) in the 9×10^–2^ mg/ml Artemisinin with iron (36^th^ hour). Invert microscopy (bar=30 μm).

Both control groups (with and without iron) did not show any pathologic changes in the microscopic tests (Figures 1 and 2). In the combination therapy groups, pathologic changes were observed from the first concentration (Table 1). In treatment groups that received Artemisinin alone, the observed lesions were somewhat slighter than in the group with combination therapy. Lesions began from higher concentrations and later hours in the Artemisinin group, but the types of lesions were similar (Figures 3–7). In the group that received 1.2 μg/ml of Artemisinin, 45% of the culture surface was filled with cells at the hour 60. This percentage was 10% for the 1.2 μg/ml combination therapy group, yet hematoxylin-eosin staining indicated that some of these cells were dead.

**Table 1:** Qualitative microscopic data in various concentrations and hours.

	HOURS OF EXPOSURE				
Groups μg/ml	12	24	36	48	60
**0.15 Fe + Artemisinin**	-------------	-------------	slight vacuolation of cells with small vacuoles	slight vacuolation of cells with small vacuoles	slight vacuolation of cells with small vacuoles
**0.3 Fe + Artemisinin**	-------------	-------------	slight vacuolation of cells with small vacuoles	moderate vacuolation of cells with small vacuoles, cells welling, nuclear swelling	moderate vacuolation of cells with small vacuoles, sell swelling, nuclear swelling, scattered piknosis
**0.6 Fe + Artemisinin**	slight vacuolation of cells with small vacuoles	slight vacuolation of cells with large vacuoles, sell swelling, nuclear swelling, slight piknosis, scattered vesicle formation	moderate vacuolation of cells with large vacuoles, cell swelling, nuclear swelling, moderate piknosis, moderate vesicle formation	severe vacuolation of cells with large vacuoles, cell swelling, nuclear swelling, moderate piknosis, moderate vesicle formation, scattered denudation of cell culture surface from cells and new mitosis around cells to fill the evacuated area	severe vacuolation of cells with large vacuoles, cell swelling, nuclear swelling, severe piknosis, severe vesicle formation, moderate denudation of cell culture surface from cells and new mitosis of around cells to fill the evacuated area
**1.2 Fe + Artemisinin**	slight vacuolation of cells with small vacuoles, slight cell and nuclear swelling	severe vacuolation of cells with large vacuoles, cell swelling, nuclear swelling, moderate piknosis, scattered vesicle formation	severe vacuolation of cells with large vacuoles, cell swelling, nuclear swelling, severe piknosis, severe vesicle formation	severe vacuolation of cells with large vacuoles, cell swelling, nuclear swelling, severe piknosis, severe vesicle formation, moderate denudation and no new mitosis	severe vacuolation of cells with large vacuoles, cell swelling, nuclear swelling, severe piknosis, severe vesicle formation, very extensive denudation no new mitosis, a few clumps of deformed necrotic cells, some karyorrhexis
**0.15 Artemisinin**	-------------	-------------	--------------	--------------	--------------
**0.3 Artemisinin**	-------------	-------------	--------------	slight vacuolation of cells with small vacuoles	slight vacuolation of cells with small vacuoles, slight cell and nuclear swelling
**0.6 Artemisinin**	-------------	-------------	slight vacuolation of cells with small vacuoles, slight cell and nuclear swelling	slight vacuolation of cells with large vacuoles, moderate cell and nuclear swelling, slight piknosis	moderate vacuolation of cells with large vacuoles, moderate cell and nuclear swelling, moderate piknosis, slight denudation and new mitosis, scattered vesicle formation
**1.2 Artemisinin**	-------------	slight vacuolation of cells with small vacuoles, slight cell and nuclear swelling, scattered piknosis	moderate vacuolation of cells with large vacuoles, moderate cell and nuclear swelling, moderate piknosis scattered vesicle formation denudation and new mitosis	extensive vacuolation of cells with large vacuoles, severe cell and nuclear swelling, severe piknosis, moderate vesicle formation denudation and new mitosis	extensive vacuolation of cells with large vacuoles, severe cell and nuclear swelling, severe piknosis, severe vesicle formation denudation and new mitosis

### Biochemistry

In the combination therapy groups, LDH and total protein levels increased in a concentration dependent manner in the supernatants and cell fraction extracts. In the cell fraction extracts, data for the TBARS test showed a concentration dependent enhancement. The increase was statistically significant from the concentration of 0.6 μg/ml (Tables 2 and 3).

**Table 2:** Supernatant biochemistry following treatment with Artemisinin and Artemisinin with iron.

	LDH U/mg pro × 10^–3^		Total protein mg/ml × 10^–1^	
	
	Artemisinin	Artimisinin + Fe	Artemisinin	Artimisinin + Fe
control	6.9±0.1	7.9±0.2	21.7±1.8	18.9±0.4
Artemisinin (0.15 μg/ml)	8.2±0.3	8.25±0.4	21.2±0.3	20.1±0.1
Artemisinin (0.3 μg/ml)	9±0.2	9±0.5	21.4±0.8	22.2±1
Artemisinin (0.6 μg/ml)	13±1.4	21±5.7	22.9±0.2	23.2±1.2
Artemisinin (1.2 μg/ml)	22.3±0.2	46±4.2	22.5±0.7	24.5±1.4

**Table 3:** Cell biochemistry following extraction of cell fractions.

	LDH U/mg pro × 10^–3^		Total protein mg/ml× 10^–1^		MDA nmol/mg pro × 10^–3^	
	Artemisinin	Artimisinin + Fe	Artimisinin	Artimisinin + Fe	Artemisinin	Artimisinin + Fe
control	210.6±0.9	238±0.2	32.5±1.5	37±1.5	4.5±0.8	4.7±0.4
Artemisinin (0.15 μg/ml)	241±9.8	252±11.2	33.9±.2	34.1±0.4	4.8±0.6	5.5±0.1
Artemisinin (0.3 μg/ml)	259.1±2.8	299±1	39.2±0.1	40.5±2	5.5±0.9	6±0.1
Artemisinin (0.6 μg/ml)	265±7	365±35.3	44.5±4	42.4±3.2	7.9±0.7	9.4±0.8
Artemisinin (1.2 μg/ml)	371.3±0.6	425±21.2	46.4±1.2	58.5±0.9	9.5±0.2	12±0.7

In the Artemisinin treatment groups, LDH was significantly different from the 0.6 μg/ml concentration within the control group. Total protein did not show any difference in the supernatants of the treatment groups compared with controls. However, in the cell fraction extracts, LDH changes were significantly different from the control group starting with the 0.15 μg/ml concentration. TBARS and other parameters were statistically different from the control in the cell fraction extracts starting with the 0.6 μg/ml concentration (Table 4).Within the two control groups, LDH was the only differing variable (*p=*0.034).Comparison of the various parameters within the combination therapy and Artemisinin treatment groups, with the same concentration of Artemisinin, indicated that all parameters were higher within the combination therapy groups and that the enhancement of the Artemisinin dosage resulted in a statistical difference for all the variables.

**Table 4:** *p*-values of the Tukey test between treatment groups and controls. Concentrations less than 0.6 μg/mL were omitted since, for all parameters, the differences were not significant.

Groups (μg/ml)	LDH (unit/mg prot.)	Total protein (mg/ml)	MDA (nmol/mg prot.)
0.6 Fe + Artemisinin supernatant	0.044	0.033	--------------------
1.2 Fe + Artemisinin supernatant	≤0.001	0.012	--------------------
0.6 Fe + Artemisinin cell fraction	0.006	Not significant	0.023
1.2 Fe + Artemisinin cell fraction	0.001	0.001	0.002
0.6 Artemisinin supernatant	0.002	Not significant	--------------------
1.2 Artemisinin supernatant	≤0.001	Not significant	--------------------
0.15 Artemisinin cell fraction	0.036	Not significant	Not significant
0.3 Artemisinin cell fraction	0.005	Not significant	Not significant
0.6 Artemisinin cell fraction	0.003	0.01	0.028
1.2 Artemisinin cell fraction	≤0.001	0.005	0.005

## Discussion

Oxidative stress is considered an important contributory mechanism in cell injury and is associated with a number of disorders, including atherosclerosis, ischemia/reperfusion injury, arthritis, stroke and neurodegenerative diseases (Thamilselvan *et al*., 2000), as well as with a variety of metabolic, toxic, or hypoxic conditions. (Thamilselvan *et al*., 2009). Additionally, reactive oxygen species (ROS) have detrimental effects on proteins, lipids, and DNA, and promote severe tissue damage and cell death (Ryter *et al*., 2007).

Intracellular reactive oxidants are generated both by enzymatic and nonenzymatic sources including the mitochondrial electron transport system, NADPH oxidase, 5-lipoxygenase, several oxidases located within subcellular compartments such as peroxidases and mono- and dioxygenases, and isoenzymes of the cytochromeP-450 superfamily which includes nitric oxide synthase, xanthine oxidase, and cyclo oxygenase (Thamilselvan *et al*., 2009).

The generation of hydrogen peroxide has been implicated in the pathogenesis of several forms of acute tubular cell injury (Baliga *et al*., 1997). Whereas several lines of evidence support the view that CL2(A kind of membrane protein channels) may contribute to cell injury induced by hypoxia or ATP depletion, the role of CL2 in oxidant-induced injury has not been determined. Additionally, even in hypoxia or ATP depletion, the role of CL2 in cell injury has not been firmly established. For example, although certain CL2 channel blockers reduce LDH release from proximal tubules or MDCK cells subjected to hypoxia or ATP depletion, the substitution of CL2 by other anions has not been shown to confer protection (Meng & Reeves, 2000).

The main Artemisinin drug family actions are considerd to be due to the endoperoxide bridge. In the body these drugs produce free oxygen and carbon centered radicals. These radicals damage the cell membrane and some vital cellular elements (Golenser *et al*., 2006). Also, Artemisinins can cause injuries to the mitochondria through the depolarization of its membrane and through the inhibition of NADH dehydrogenase (Kamchonwongpaisan *et al*., 1997; Zhao & Zhuang, 1987). The use of this drug family in some humans was reported to cause urine darkness and discoloration. The intravenous administration of Artesunate and Artelinate in rats was observed to cause urinary problem related to slight to moderate tubular necrosis. The injury was reversed eight days following usage of the drug (Li & Peggins, 1998). The oral administration of Artemether caused an enhancement of blood urea (Shuhua *et al*., 2002). Our previous work showed the presence of eosinophilic intra-cytoplasmic inclusion bodies and some eosinophilic droplets in the lumen of the renal tubules in broiler chickens (Arab *et al*., 2009; Shahbazfar *et al*., 2011), evidence of renal tubular degeneration (Randall & Reece, 1996).

Lipid peroxidation indicates oxidative tissue damage by hydrogen peroxide, superoxide, and hydroxyl radicals resulting in structural alteration of membranes with the release of cell and organelle content, loss of essential fatty acids, and formation of cytosolic aldehyde and peroxide products (Thamilselvan *et al*., 2000). Malonyl dialdehyde is a major end product of the free radical reaction with membrane fatty acids (Thamilselvan *et al*., 2000).

The exposure of MDCK cells to Artimisinin has also caused an increase in malonyl dialdehyde (TBARS test). The result is similar to the effect of oxalate and calcium oxalate monohydrate on MDCK cells (Thamilselvan *et al*., 2000), and these effects were found to be mediated by free radicals. We propose that NADPH oxidase-derived free radicals play a prominent role in renal injury in the presence of Artemisinin. One pathway of injury to epithelial cells is through the role of RAC GTPase **(**Rac is a subfamily of the Rho family of GTPases), (Thamilselvan *et al*., 2011). Members of the Rho family of Ras-related small GTPases play a central role in the control of actin dynamics, cell migration, and other cellular processes. Rac isoforms are involved in membrane ruffling and lamellipodial protrusion (Burridge & Wennerberg, 2004).

The exposure of MDCK cells to Artimisinin caused a noticeable increase in LDH. The release of LDH is also a marker of cell injury (Thamilselvan, 2011). In the course of hypoxic injury to proximal tubules, cell membranes first become permeable to small molecules, then progressively to larger molecules and macromolecules (Chen & Mandel, 1997). Indeed, the development of membrane permeability to macromolecules, such as lactate dehydrogenase, has been considered a marker of irreversible necrotic cell death (Thamilselvan *et al*., 2011). Some studies have also implicated size selectivity of the plasma membrane during chemical ATP depletion in MDCK cells. After two hours of ATP depletion, cell membranes became openly permeable to dextran with a size of 4 kDa, partly permeable to 70-kDa dextran, and only slightly permeable to dextrans larger than 145 kDa (Dong *et al*., 1998).

Cell and nuclear swelling was observed as one of the early changes in our study. The maintenance of cellular ion homeostasis requires a precise balance of solute influx and efflux via both active and passive membrane transport pathways. Some authors have proposed that the uptake of a solute, mainly sodium and chloride, leads within cells to cell swelling and contributes to membrane injury in renal epithelial cells and other tissues (Meng & Reeves, 2000). Cell swelling induced by the osmotic gradients produced by LDH release has been observed only at extremes in cell volume. Additionally, glycine was found to prevente LDH release from ATP-depleted MDCK cells without preventing cell swelling (Meng & Reeves, 2000).

Morphological studies suggest alterations in cell-to-cell contact in areas of cell loss and the new mitosis of viable cells in order to fill denuded areas. This mitosis is however lower or absent for higher concentrations of Artemisinin, especially in the presence of iron. Microscopic changes indicate a karyotoxic effect of the drug and mitosis appears to be disturbed and blocked at higher concentrations, so that chromosomes lie irregularly clustered in the nucleus in some, but not all, cells. A significant decrease in viability occurred also in the remaining monolayer cells. In this work, these processes were concentration and time dependent.

Biochemical indicators for cell damage were supported by morphological studies, as profound cellular alterations were demonstrated by hematoxylin-eosin staining and light microscopy. A summary of the findings supports the contention that the exposure of MDCK cells to Artemisinin results in cell damage. The results are meaningful because MDCK cells (distal tubular origin) are relatively resistant to injury, at least in comparison to cells with a proximal tubular origin, like the LLC-PK_1_ cell line (Hackett *et al*., 1994).

LDH enzyme and malonyl dialdehyde levels were considerably higher for the combined therapy with iron, and more important by then, were found to occur at an earlier time, indicating that the injuries were more severe with iron. The result is similar to other findings that showed that the addition of iron or hemin to nervous system cell cultures enhanced the injury to these cells, and, vice-versa, that the presence of ascorbic acid within cultures reduced the toxicity by decreasing oxidative stress (Smith *et al*., 1998Smith *et al*., 1999).

During the early stages, vacuolation of cells with small vacuoles can result due to granular degeneration and depletion of cells from glycogen, which is one of the early signs of degeneration. Vacuolation is followed by large vacuoles, which can be fat vacuoles, another and more progressive sign of degeneration.

The *in vitro* formation of cysts observed in this study was reported before in human renal cells and MDCK cells. These cells develop spherical monolayer cysts, which are fluid-filled structures, when cultivated in a collagen type I matrix (Pey *et al*., 1996). The MDCK cell line has been extensively used as an analogue model of pathological cyst formation in studies investigating the establishment of cell polarity, cell-matrix interactions, and transepithelial transport (Pey *et al*., 1996).

We believe that Artemisinin, as a herbal based drug, can be used for Leishmaniasis treatment in dogs, but that its use requires further study regarding the influence of the drug on the liver, as the main metabolizing organ, and the nervous system, as the organ most influenced by this drug family. Overall, the drug does have some side effects and the belief that it is completely safe since it is a herbal medication is not true. Its use thus necessitates caution and supervision.
